# Kinesitherapy for idiopathic facial palsy: A protocol of systematic review and meta-analysis

**DOI:** 10.1097/MD.0000000000023902

**Published:** 2020-12-24

**Authors:** Qiang Zhang, Chan Zhu, Jing Liu

**Affiliations:** a Sichuan Provincial Orthopedic Hospital; b Hospital of Chengdu University of Traditional Chinese Medicine; c Sichuan Bayi Rehabilitation Center, Sichuan Provincial Rehabilitation Hospital, Chengdu, Sichuan Province, PR China.

**Keywords:** Bells palsy, exercise, protocol, systematic review, therapy

## Abstract

**Background:**

Idiopathic facial nerve palsy (Bells palsy) is the most common acute mono-neuropathy which lack of effective treatments. Kinesitherapy as an alternative therapeutic approach was widely used in clinical. But the effects on Bells palsy outcome are still debated.

**Methods:**

The aim of this study is to systematically review the therapeutic efficacy of kinesitherapy for Bells palsy. Database including PubMed, EMBASE, Cochrane Library, Chinese Biomedical database (CBM), Chinese National Knowledge Infrastructure (CNKI), Chinese Science and Technology Periodical database (VIP) and WangFang database will be searched to collect randomized controlled trails (RCTs) on kinesitherapy for Bells palsy from inception to Nov 2020. The therapeutic effects based on recovery rate, House-Brackmann (H-B) facial classification system, Sunny brook face grading system and adverse events after the treatment will be marked as the primary outcomes. RevMan V.5.3 software will be used to calculate the data synthesis as well as to perform meta-analysis if the results are appropriate.

**Results:**

The literature will provide a high-quality synthesis of current evidence of kinesitherapy for Bells palsy from various comprehensive assessment, including the recovery rate, H-B facial classification, Sunny brook face scores, adverse events rate, Facial disability index (FDI), residual symptoms 6 months after onset, incomplete recovery rate after 1 year.

**Conclusion:**

The systematic review will provide up-to-date evidence to assess kinesitherapy for Bells palsy.

**PROSPERO registration number:**

PROSPERO CRD42020215109.

## 1 Introduction

Idiopathic facial nerve palsy (Bells palsy) is a common acute mono-neuropathy, with incidence ranging from 11.5 to 53.3 per 100,000 person years in different populations.^[1]^ The major cause of Bells palsy is believed to be the facial nerve was infected by the herpes simplex virus.^[2]^ As a result of this, the facial nerve swells and is compressed in its canal as it courses through the temporal bone. Most patients with Bells palsy show some recovery without intervention within 2 to 3 weeks after onset of symptoms and completely recover within 3 to 4 months.^[3]^ Though the paralysis will self-limited most of time, additional long-term poor outcomes do occur. Residual deficits including facial asymmetry; hemifacial spasm; abnormal lacrimation with eating, known as Bogorad syndrome (crocodile tears); corneal ulceration and permanent visual impairment. The incidence of Bells palsy was noted to be highest in 15- to 45-year-old patient group,^[3]^ hence, the psychological burden can be tremendous for them.^[4]^

Currently, medical therapy (steroids and antivirals, alone and in combination) is the main option among the myriad treatments. However, these strategies cannot obtain a desired satisfaction. Surgical decompression was debated for the unexpected risks.^[5]^ Notably, complementary therapies were widely used in clinical, known as less side effects but lack of high quality evidence to verify the effects.^[6,7]^

According to the best to our knowledge, we found only 1 prior systematic review^[6]^ referred to the efficacy of kinesitherapy (tailored facial exercise) for Bells palsy and noted that “there is low quality evidence that tailored facial exercises can help to improve facial function, mainly for people with moderate paralysis and chronic cases. There is low quality evidence that facial exercise reduces sequelae in acute cases.” Comparing to the other physical therapies, kinesitherapy seemed promising. However, the researchers did not update since 2011. Therefore, we are aiming to make a specific systematic review to identify the efficacy and safety of kinesitherapy for Bells palsy with high-quality and large sample evidence.

## 2 Methods

### 2.1 Inclusion criteria for study selection

#### 2.1.1 Types of studies

All the relevant randomized controlled trials (RCTs) conducting kinesitherapy for the treatment of Bells palsy will be included without consideration of using blind method or not. Quasi-randomized and observational studies will be excluded. Language limitation: English and Chinese.

#### 2.1.2 Types of patients

Participants, patients who were clinically diagnosed with idiopathic facial nerve palsy, according to the Guideline on the Diagnosis and Treatment of Idiopathic facial nerve palsy,^[1,8]^ will be included. Gender and race will not be considered.

#### 2.1.3 Types of interventions

The intervention in eligible studies of interest is the experimental group, which will be treated with standard procedures (SPs) and kinesitherapy, while the control group will receive the same SP (e.g., oral steroids, oral antiviral therapy, eye protection).

#### 2.1.4 Types of outcome measures

##### 2.1.4.1 Major outcomes

- Recovery rate;
- H-B facial classification;
- Sunny brook face scores;
- Adverse events.

##### 2.1.4.2 Secondary outcomes

- Residual symptoms 6 months after onset, such as motor synapse, contracture, hyperactivity, facial spasm or crocodile tears;
- Incomplete recovery after 1 year;
- FDI.

### 2.2 Search methods for the identification of studies

#### 2.2.1 Electronic searches

PubMed, EMBASE, Cochrane Library, Chinese Biomedical database (CBM), Chinese National Knowledge Infrastructure (CNKI), Chinese Science and Technology Periodical database (VIP) and WangFang database will be searched to from inception to Nov 2020 for the RCTs of kinesitherapy for Bells palsy. On the basis of Cochrane handbook, detailed strategies for searching the PubMed database in Supplemental Digital Content (Appendix A), and similar strategies will be applied to remaining databases.

#### 2.2.2 Searching other resources

We will manually review the references lists of included studies and previous systematic reviews for other potentially relevant literatures. Also, conference proceedings for the American Academy of Neurology (AAN), the American Academy of Otolaryngology-Head and Neck Surgery Foundation (AAO-HNSF), Canadian Society of Otolaryngology-Head and Neck Surgery and Canadian Neurological Sciences Federation will be retrieved in last 5 years. What is more, we will contract experts in the field to see if they are aware of other studies on this topic.

### 2.3 Data collection and analysis

#### 2.3.1 Selection of studies

**Figure 1:**
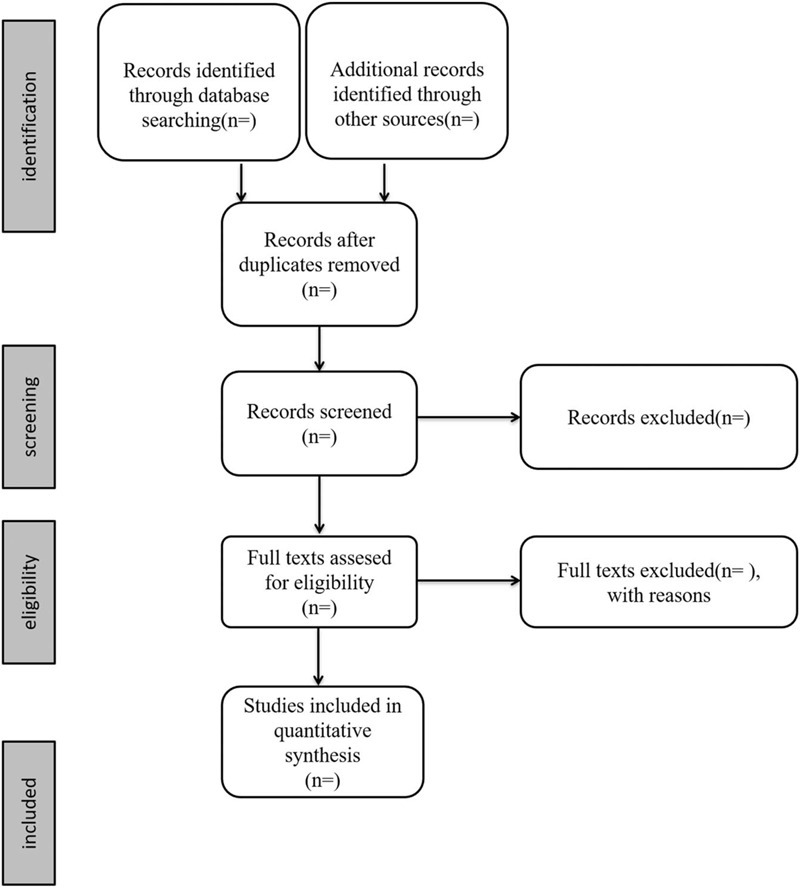
Flow diagram of study selection process.

Two experienced authors will first search all databases independently to screen the titles and abstracts of relevant studies removing duplications. Another 2 independent researchers will review all the extracted titles and abstracts, and screen full texts based on the previous inclusion criteria to identify eligible studies. Removed studies after full text review will be recorded with specific exclusion reason. Any disagreement will be solved by group discussion. If necessary, the third reviewer will be consulted. The selection process of eligible papers is shown in a Preferred Reporting Items for Systematic Review and Meta-analysis (PRISMA) flow diagram (Fig. 1).

#### 2.3.2 Data collection and management

Two reviewers will conduct collection independently using prepared extraction forms. Data extraction will include study characteristics, population characteristics, interventions of the experimental and control groups, major data for the focus outcomes, as well as relevant indicators of bias risk assessment.

#### 2.3.3 Assessment of risk of bias in included studies

Cochrane Collaboration Tool ^[9]^ will be adopted to the assessment of risk of bias. Two researchers will independently be in charge of evaluation for each included study. Each original research will be assessed in 6 domains: random sequence generation, allocation concealment, blinding of participants and personnel, blinding of outcome assessment, selective reporting and other sources of bias, and classified as “low”, “high”, or “unclear” according to the tool. If there is any disagreement in procession, we will reach a consensus through discussion or consult with a senior author.

#### 2.3.4 Measures of treatment effect

Using RevMan 5.3 [Review Manager (RevMan) (Computer program), Version 5.3, Copenhagen: The Nordic Cochrane Center, The Cochrane Collaboration, 2014], we will perform a meta-analysis if the results are appropriate. For dichotomous outcomes, the extracted data will be presented as rate ratio (RR) with 95% confidence interval (95% CI). As for continuous data, results will be computed as mean difference (MD) with 95% CI.

#### 2.3.5 Dealing with missing data

If required information is not available in included literatures, the authors will connect with the corresponding author of the primary studies by E-mail for complete information. If no additional message is received, we will conduct data synthesis suing available data. But at the same time, we will also discuss the possible consequence the missing data might cause in the review.

#### 2.3.6 Assessment of heterogeneity

Heterogeneity among trails will be undertaken to evaluate the feasibility of meta-analysis. If *I*^*2*^ value is over 50%, we will consider the significant heterogeneity and perform subgroup analysis to investigate the potential cause from clinical or methodological heterogeneity.

#### 2.3.7 Assessment of reporting bias

If figure of included trails is adequate (over 10 pieces) in the review, we will put funnel plot according to Egger methods to discuss the reporting bias or small-study effects.

#### 2.3.8 Data synthesis

RevMan software will be computed to calculate for data synthesis when a meta-analysis is suitable. On the condition that no obvious statistical heterogeneity is found among the trails, fixed effects model will be performed in the analysis. If not, the reviewers will detect the source of statistical heterogeneity in further analysis. However, if apparent clinical heterogeneity is exploded, then the random effects model will be employed. At the same time, subgroup or sensitivity analysis will be carried out. α = 0.05 will be deemed statistical significant. If it is not available to conduct a meta-analysis, we will only describe the data.

#### 2.3.9 Subgroup analysis

Subgroup analysis will be carried out in the premise of sufficient eligible studies (at least 10 trails). Exploring the resources of the heterogeneity, we will assess inconsistent participants characteristics, classification of Bells palsy, basic diseases, premonitory symptom, dose of taking medicine, and other unpredictable factors.

#### 2.3.10 Sensitivity analysis

Sensitivity analysis will be adopted to detect the quality of included studies of the document following sample size, the outcome of missing data, and methodological quality.

#### 2.3.11 Ethics and dissemination

The results of the systematic review will be disseminated via publication in a peer-reviewed journal and presented at a relevant conference. The data we will use do not include individual patient data, thus, ethical approval is not required.

## 3 Discussion

Bells palsy is very common in outpatient. There are several known risk factors for Bells palsy, including pregnancy,^[10]^ severe preeclampsia, obesity, hypertension, diabetes and upper respiratory ailments.^[1]^ For those patients, medication might be a double-edged sword. Kinesitherapy, as a non-invasive alternative therapy was widely used in clinical practice. Our previous RCT study^[11]^ disclosed that kinesitherapy did has certain effect on Bells palsy. It cannot be denied that plenty of clinical studies on this topic were published since 2011; therefore, we begin to conduct the systematic review and meta-analysis of existing studies to objectively evaluate the feasibility and rationality of kinesitherapy in the treatment of patients with Bells palsy. All the trails included will be performed in accordance of Cochrane Handbook to ensure the provided helpful information for clinicians and Bells palsy patients.

## Author contributions

**Conceptualization:** Chan Zhu.

**Data curation:** Qiang Zhang.

**Formal analysis:** Qiang Zhang, Chan Zhu, Jing Liu.

**Funding acquisition:** Qiang Zhang.

**Resources:** Jing Liu.

**Software:** Qiang Zhang.

**Writing – original draft:** Qiang Zhang.

## Supplementary Material

Supplemental Digital Content
