# Supplementary figures and images for: Kinesitherapy for idiopathic facial palsy: A protocol of systematic review and meta-analysis
Source: Medicine (Baltimore). 2020 Dec 24;99(52):e23902. doi: 10.1097/MD.0000000000023902 (PMC7769292; doi:10.1097/MD.0000000000023902)

**Appendix A.** search strategy of kinesitherapy for idiopathic facial nerve palsy


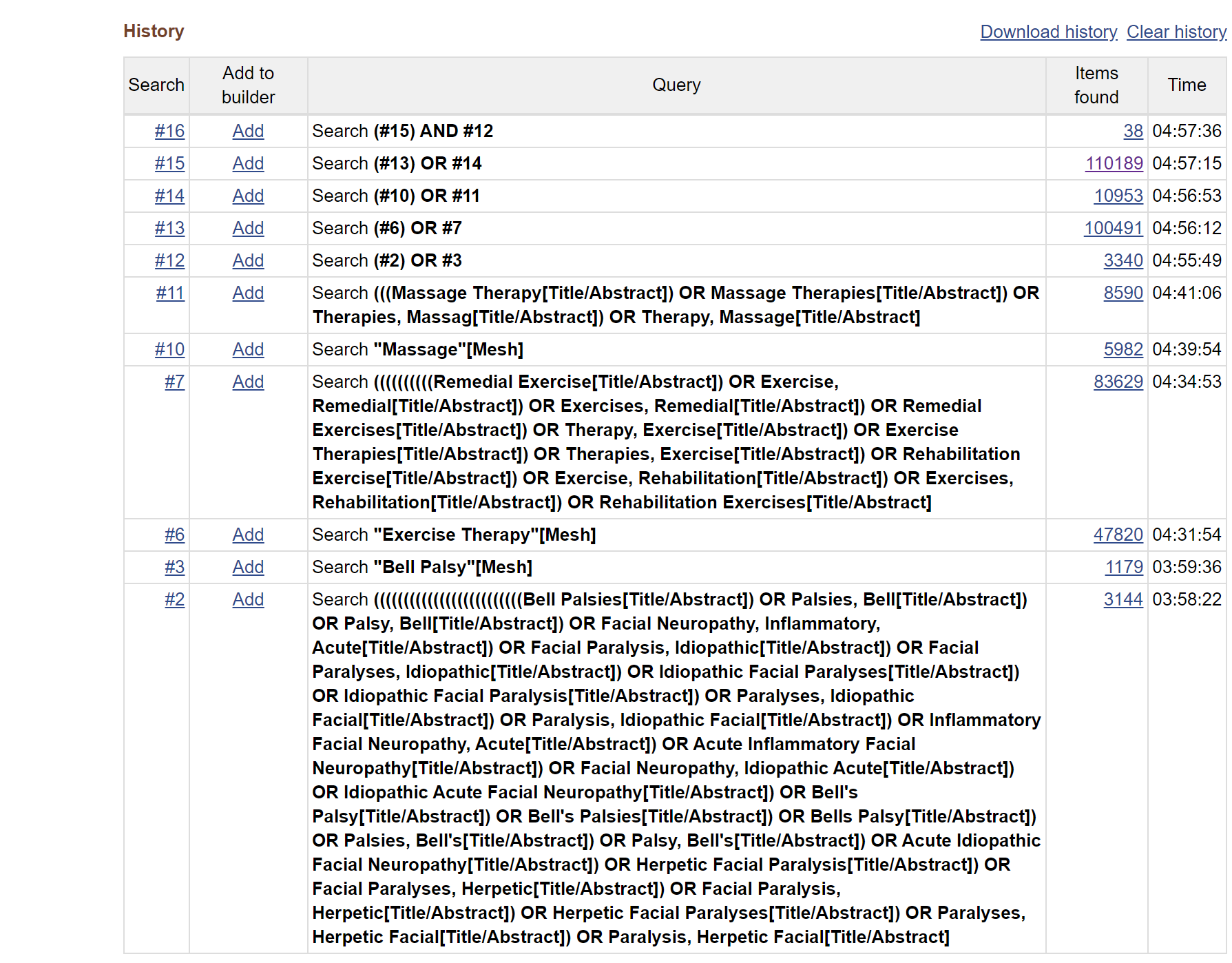

Supplement: Supplemental Digital Content [file medi-99-e23902-s001.docx]
